# The role of *Akkermansia muciniphila* in the regulation of inflammatory bowel disease: intestinal immunity and metabolism

**DOI:** 10.3389/fimmu.2025.1653472

**Published:** 2025-12-19

**Authors:** Wang Xu, Aijing Li, Huijuan Jing, Xiaoping Zhang, Xucheng Dong, Zhiqiang Song, Nanping Wu, Shufa Zheng

**Affiliations:** 1 Jinan Microecological Biomedicine Shandong Laboratory, Jinan, China; 2 State Key Laboratory for Diagnosis and Treatment of Infectious Diseases, National Clinical Research Center for Infectious Diseases, National Medical Center for Infectious Diseases, Collaborative Innovation Center for Diagnosis and Treatment of Infectious Diseases, The First Affiliated Hospital, Zhejiang University School of Medicine, Hangzhou, China

**Keywords:** *Akkermansia muciniphila*, inflammatory bowel disease, gut microbiota, intestinal immunity, immune cells, metabolism

## Abstract

Inflammatory bowel disease (IBD) is closely associated with the abundance of *Akkermansia muciniphila* (*A. muciniphila*), a resident member of the intestinal tract that is being developed as a next-generation probiotic. Accumulated evidence has indicated that the live and pasteurized *A. muciniphila*, as well as its components and secretions, have exhibited protective and ameliorative functions in IBD. Nevertheless, the precise and intricate regulatory mechanisms of *A. muciniphila* in IBD remain unclear, which is crucial for investigating the etiology of IBD and searching for innovative, targeted therapeutic strategies. In this review, we discuss the reciprocal influence between *A. muciniphila* and intestinal immunity in IBD, encompassing the roles of immune cells, intestinal epithelial cells (IECs), and intestinal stem cells (ISCs). Subsequently, we outline the mutual regulatory interactions between *A. muciniphila* and intestinal metabolism, focusing on tryptophan (Trp) metabolism, short-chain fatty acids (SCFAs) metabolism, and bile acids (BAs) metabolism. Understanding how *A. muciniphila* interacts with its host is a vital step for facilitating its application in IBD therapy.

## 1 Introduction

**Figure 1:**
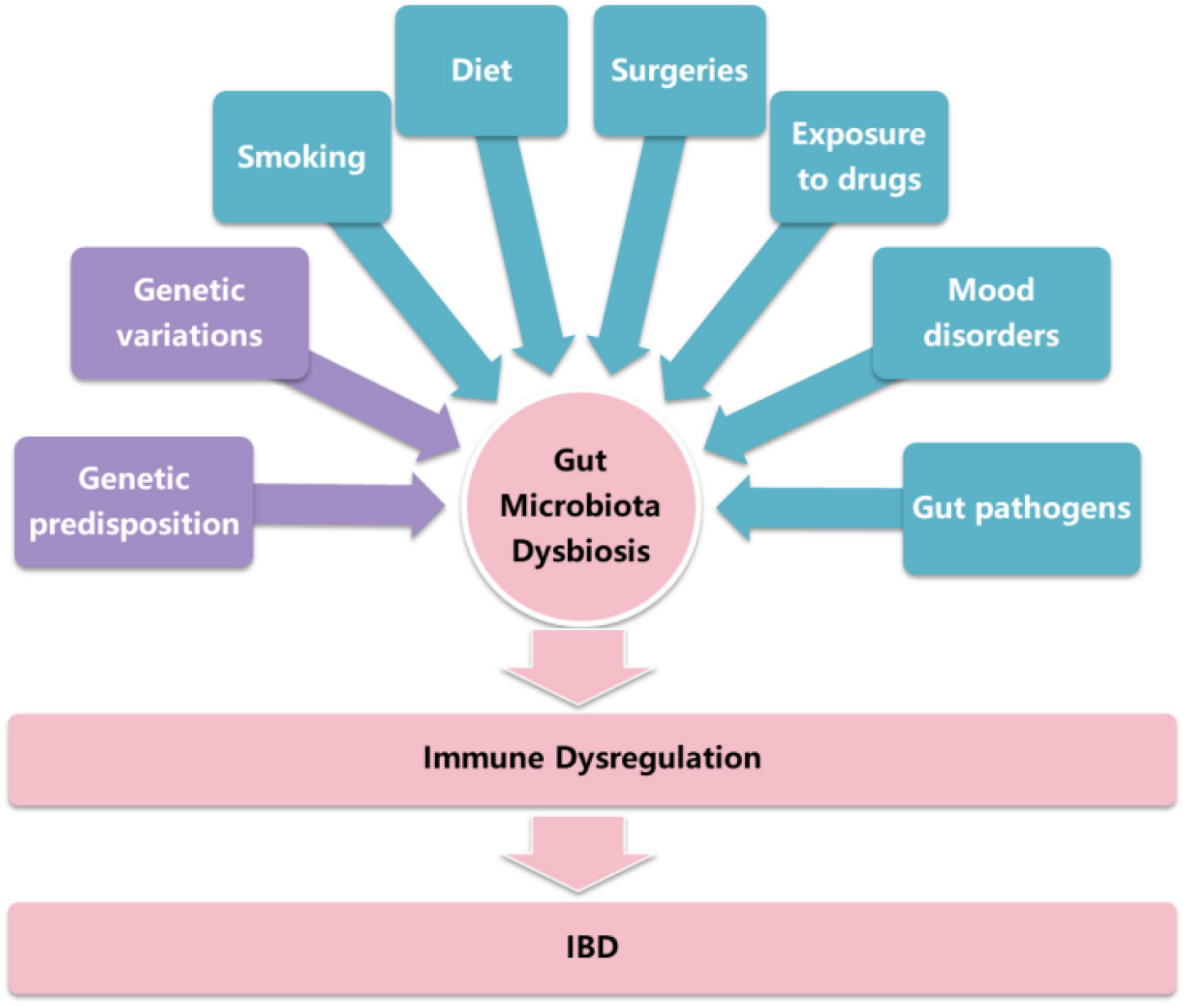
The factors involved in the pathogenesis of IBD.

The IBD, including Crohn’s disease (CD) and ulcerative colitis (UC), is a chronic inflammatory disease driven by inappropriate intestinal immune activation. The etiology of IBD is complex and multifactorial, primarily involving genetic and environmental factors, which perturb the homeostasis between the gut microbiome and the host immune system (**Figure 1**) (1, 2). The burden of IBD across the globe is still considerable (3). Patients with IBD have an increased risk of colorectal cancer, which is the third most common cancer in the world (9.6% of all cancers globally) (4–6). Conventional IBD treatment methods involve medication therapy, e.g., aminosalicylates, corticosteroids, antibiotics, biological agents, small molecule drugs, and surgical treatment if necessary (7). Researchers have identified that patients with IBD exhibit altered bacterial diversity and abundance compared to healthy individuals. In addition, the Gram-negative bacteria exhibit the main difference in fecal microbiota between UC patients and healthy individuals (8). The precise mechanisms of host-microbiota crosstalk in IBD remain incompletely elucidated. To address this, multi-omics approaches are being leveraged to systematically decode these complex cross-talk networks (9–13). Consequently, modulating the gut microbiota has emerged as a promising strategy for restoring homeostasis and advancing novel therapeutic interventions for IBD (8, 14, 15).

*A. muciniphila* is a species of the genus *Akkermansia*, which belongs to the family *Akkermansiaceae* in the phylum *Verrucomicrobiota*. It was initially isolated from healthy female feces in 2004 and cultivated in a specific medium containing mucin as the sole carbon and nitrogen source (16, 17). *A. muciniphila* is a Gram-negative commensal bacterium that is dominantly distributed in the intestinal mucus layer and fecal samples of both human and animals. Its relative abundance is approximately 1-4% (10^6–^10^8^ CFU/g) of total bacteria in feces of healthy adults (18, 19). *A. muciniphila* in the intestinal tract is known as a mucolytic specialist, primarily degrading mucins (20, 21). This process not only leads to the renewal of mucins but also results in the release of oligosaccharides and short-chain fatty acids (SCFAs), which all play an important role in microbial community and host health (16, 22–27).

Several studies have demonstrated that the abundance of *A. muciniphila* is significantly altered in both IBD patients and model mice compared with healthy controls (28–30). Recent studies have demonstrated that the gut microbiota composition in UC patients during long-term remission closely resembles that of healthy individuals. Notably, the abundance of *A. muciniphila* increases significantly during the remission phase compared to the active phase of CD (31, 32). The live and pasteurized *A. muciniphila*, as well as its components, e.g., outer membrane proteins, extracellular vesicles, have been evidenced to be relevant to IBD, which predominantly influence the immune responses, gut microbiota, metabolism, and the integrity of the intestinal barrier (30, 33–37). However, the role of *A. muciniphila* in IBD remains contentious, as it may exert pro-inflammatory effects under specific conditions. These include bacterial strain differences; host species disparities (e.g., human, mouse); the presence of pathogen-induced inflammation (such as *Salmonella Typhimurium* infection); reconstitution of the gut microbiota following antibiotic treatment; and increased susceptibility in certain hosts. Such susceptible individuals may include those with polycystic ovary syndrome (PCOS), endometriosis, disrupted gastrointestinal motility, as well as people carrying genetic defects (e.g., IL-10 or HNF4A deficiency) (38–42).

Although *A. muciniphila* has shown promise in the intervention of IBD, its specific mechanisms of action remain complex and unexplained. In this review, we outline the interaction of *A. muciniphila* in IBD with intestinal immunity and gut metabolism. These interactions are crucial for modulating intestinal immune responses, the integrity of the intestinal barrier, the homeostasis of intestinal microbiota, and metabolism.

## 2 The *A. muciniphila* and intestinal immunity

**Figure 2:**
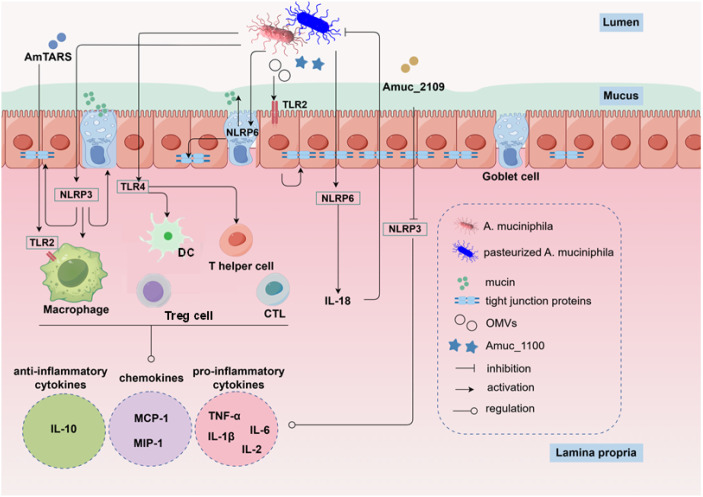
Live and pasteurized *A. muciniphila*, along with its components (Amuc_1100) and secreted products (AmTARS, OMVs), can activate TLRs and NLRPs on immune cells and IECs to ameliorate IBD. This action proceeds via two primary mechanisms. First, it modulates intestinal immunity by promoting the release of anti-inflammatory cytokines (IL-10) and suppressing pro-inflammatory cytokines (e.g., TNF-α, IL-1β, IL-6). Additionally, it inhibits the production of pro-inflammatory chemokines (e.g., MCP-1, MIP-1), which are key drivers of excessive monocyte/macrophage activation and inflammation. Second, it enhances the intestinal barrier by stimulating mucin secretion from goblet cells and increasing the expression of tight junction proteins in IECs. Acronyms in the figure: AmTARS: *A. muciniphila* secreted threonyl-tRNA synthetase; Amuc_2109: *A. muciniphila* secreted β-N-acetylhexosaminidase; Amuc_1100: an outer membrane protein of *A. muciniphila*; OMVs: *A. muciniphila* derived outer membrane vesicles; TLR2: toll-like receptor 2; TLR4: toll-like receptor 4; DC: dendritic cell; CTL: cytotoxic T lymphocyte; NLRP6: NOD-like receptor thermal protein domain associated protein 6; NLRP3: NOD-like receptor thermal protein domain associated protein 3; MCP-1: monocyte chemotactic protein-1; MIP-1: macrophage inflammatory protein-1; IL-10: interleukin-10; IL-1β: interleukin-1β; IL-6: interleukin-6; IL-18: interleukin-18; TNF-α: tumor necrosis factor-α.

The development of IBD is mainly associated with impaired intestinal health, which is primarily characterized by immunological imbalance, microbiota dysbiosis, and impaired intestinal barrier function. The maintenance of intestinal health in mammals deeply relies on various types of intestinal cells, dominantly including immune cells residing in the intestinal lamina propria, intestinal epithelial cells (IECs), and intestinal stem cells (ISCs). Several insights from the microbiome indicate that *A. muciniphila* plays a pivotal role in shaping the development and function of intestinal cells. Various studies suggested that *A. muciniphila* is essential for influencing the development and function of intestinal cells in IBD (**Figure 2**).

### 2.1 The *A. muciniphila* and intestinal immune cells

The immune system comprises innate and adaptive branches, linked by dendritic cells (DCs) as a critical connection. The adaptive immune response is mainly mediated by lymphocyte receptors that recognize specific protein antigens, whereas the innate immune response is predominantly activated through pattern recognition receptors (PRRs) that are commonly expressed on innate immune cells. The PRRs involved in the innate immune response mainly contain transmembrane proteins, e.g., Toll-like receptors (TLRs), C-type lectin receptors, and cytoplasmic proteins, e.g., NOD-like receptors (NLRs), retinoic acid-inducible gene-I-like receptors (39, 43, 44).

#### 2.1.1 The *A. muciniphila* and TLRs

TLRs are a class of transmembrane proteins located on the cell membrane and endosome membrane. Signal transduction initiated by TLRs ultimately induces the secretion of diverse immune mediators. These include inflammatory cytokines, e.g., tumour necrosis factor-α (TNF-α), interleukin-1β (IL-1β), interleukin-6 (IL-6), interleukin-10 (IL-10); chemokines, e.g., monocyte chemotactic protein-1 (MCP-1), macrophage inflammatory protein-1 (MIP-1); as well as major histocompatibility and costimulatory molecules. These components are essential for the immune defense against infections and inflammatory diseases (45–47).

Several components of *A. muciniphila* have been shown to interact with TLRs, particularly TLR2 and TLR4, and subsequently contribute to the amelioration of inflammation in colitis mice. Ottman et al. cultivated and stimulated the human HEK-Blue reporter cell lines that expressed TLR2, TLR4, TLR5, TLR9, or NOD2 receptors with live *A. muciniphila*, bacterial fractions, and lipopolysaccharide (LPS) from *A. muciniphila*. The live *A. muciniphila* effectively triggered TLR2 or TLR4-mediated NF-κB activation. Further investigation revealed that pili-like protein Amuc_1100, which localized at the outer membrane of *A. muciniphila*, exhibited the ability to induce TLR2 activation and produce IL-1β, IL-6, IL-8, IL-10, and TNF-α in peripheral blood mononuclear cells (PBMCs) (48). Research shows that the lipooligosaccharide (LOS) from *A. muciniphila* could also activate both TLR4 and TLR2 (49). Kin et al. demonstrated that the threonyl-tRNA synthetase secreted from commensal bacterium *A. muciniphila* (AmTARS) could ameliorate dextran sulfate sodium (DSS)-induced colitis through directly targeting TLR2 in intestinal macrophages. TLR2 in macrophages initiated the mitogen-activated protein kinase (MAPK) and the phosphoinositide 3-kinases (PI3K)/protein kinase B (AKT) pathways, which inhibited the activity of GSK3β through AKT-mediated phosphorylation of GSK3β at Ser9. This process led to an enhanced interaction between the coactivator cAMP response element-binding protein (CREB) and CREB-binding protein (CBP), which ultimately promoted anti-inflammatory cytokines IL-10 production and suppressed the release of pro-inflammatory cytokines (50).

TLR4-deficient (TLR4^-/-^) mice manifest a heightened susceptibility to DSS-induced colitis, characterized by the dysbiosis of intestinal microbiota and disruption of immune homeostasis. Liu et al. demonstrated that the reduced levels of *A. muciniphila* and a lower frequency of suppressive RORγt^+^ Treg cells in TLR4^-/-^ mice contributed to the increased susceptibility to colon inflammation. After supplementing *A. muciniphila*, the frequency of colonic RORγt^+^ Treg cells in TLR4^-/-^ mice was increased, and the colon inflammation was suppressed. Through genomic and proteomic analysis as well as molecular modeling simulation, it was hypothesized that TLR4 plays a crucial role in influencing the intestinal colonization of *A. muciniphila*, depending on the interaction between TLR4 and the outer membrane protein Amuc_1100 of *A. muciniphila* (51).

#### 2.1.2 The *A. muciniphila* and NLRs

NLRs are mainly expressed in innate immune cells, with notable expression in IECs and lower levels observed in adaptive immune cells. The NLRP subfamily (NLPR1-14), known for having a pyrin domain, emerges as the most extensive subgroup of NLRs (44, 52). The inflammasome is an oligomeric protein complex that comprises an NLR molecule that determines the diversity of the inflammasome, a coupling molecule, and an effector molecule for cleaving the pro-interleukin (e.g., pro-IL-1β, pro-IL-18) to their mature forms (IL-1β, IL-18) (44, 53). The NLRP3 and NLRP6 inflammasomes are gaining special attention in the effects of *A. muciniphila* within IBD.

Researchers have found that the pro-inflammatory effect of *A. muciniphila* on IL-10^-/-^ susceptible mice is time-dependent. López-Cauce et al. revealed that the IL-10^-/-^ mice exhibited altered microbiota, with a significant decrease in *A. muciniphila* at week 5. Then exhibited higher intestinal permeability from week 10 and histological inflammation at week 20 (54). The research conducted by Ring et al. revealed that the *A. muciniphila* strain ATCC BAA-835 showed no signs of promoting intestinal inflammation in germ-free IL-10^-/-^ mice and SIHUMI IL-10^-/-^ mice within three weeks (55). Another study by Seregin et al. has shown that oral gavage of *A. muciniphila* for over 7 weeks could induce colitis in germ-free and SPF IL-10^-/-^ mice. But the NLRP6 played a crucial role in protecting IL-10^-/-^ mice from colitis through restricting the colonization of *A. muciniphila* in an IL-18-dependent manner. They also noted that fecal lipocalin, calprotectin, and cytokine levels exhibited no significant elevation between germ-free wild-type and germ-free NLRP6^-/-^ mice. However, the relative abundance of *A. muciniphila* was increased in NLRP6 deficiency (56). Altogether, these results suggest that the inflammation phenotype observed during the spontaneous inflammation in IL-10^-/-^ mice is secondary to the dysbiosis of gut microbiota and, particularly, the reduction in *A. muciniphila* levels. In addition, the short-term colonization of *A. muciniphila* does not cause inflammation in IL-10^-/-^ mice, but extended colonization worsens inflammation. Notably, the activation of NLRP6 inflammasomes could mitigate the onset of inflammation. Conversely, the colonization of *A. muciniphila* could stimulate the expression of NLRP6 inflammasome and autophagy-related proteins, which maintained the secretory function of Paneth and goblet cells and enhanced the mucosal barrier (57, 58).

The pretreatment with *A. muciniphila* alleviated the phenotype of DSS-induced colitis mice through activating NLRP3 and decreasing the expression of pro-inflammatory cytokines (IL1β, IL-6) and chemokines MCP-1. In addition, NLRP3 knockout (NLRP3^-/-^) mice eliminated the protective effect of *A. muciniphila* in DSS-induced colitis. Together, this study described that *A. muciniphila* plays a protective role in colitis through activating the NLRP3 inflammasome for inducing protective immunity (59).

A study discovered that β-N-acetylhexosaminidase (Amuc_2109), the secretion of *A. muciniphila*, improved the symptoms of DSS-induced colitis mice by inhibiting the activation of NLRP3 inflammasomes, and inhibited the subsequent generation of pro-inflammatory cytokines (TNF-α, IL-1β, IL-6) of mouse colonic tissues. Interestingly, the anti-inflammatory effects were entirely abolished after inactivation of Amuc_2109 (34).

While *A. muciniphila* treatment in IBD model mice exhibits the potential to alleviate inflammation through the NLRP3 inflammasome pathway, the activation or inhibition of the NLRP3 inflammasome by *A. muciniphila* or its component is currently a matter of contention.

There are some new targets of *A. muciniphila* in immune cells. The histone deacetylase 5 (HDAC5) plays a strong role in regulating the pro-inflammatory response of macrophage phenotypic polarization. Miao et al. discovered that *A. muciniphila* can inhibit HDAC5-mediated H3K9ac deacetylation, which enhances the expression of disabled homolog 2 (DAB2). This process blocks the pro-inflammatory polarization of macrophages and improves colitis (60).

#### 2.1.3 The *A. muciniphila* and DCs

As pivotal sentinels, DCs bridge innate and adaptive immunity with their capacity to induce either immune activation or tolerance, which is determined by their subtype and maturation status. A key tolerogenic mechanism is the secretion of IL-10, which fosters Treg development and Th2 responses while suppressing pro-inflammatory cytokine production. Accumulating evidence has revealed that *A. muciniphila* could ameliorate IBD through its regulatory effects on DCs. The treatment of DCs with *A. muciniphila* or its OMVs *in vitro* led to induce tolerogenic DCs, increasing the anti-inflammatory cytokine (i.e., IL-10) levels, and reducing the concentrations of pro-inflammatory cytokine (i.e., IL-12) (61). Furthermore, the OMVs can enter Peyer’s patches to elicit localized immune responses. This process involves the activation of DCs and the differentiation of B cells into plasma cells, thereby enhancing mucosal IgA production. Concurrently, OMVs reshape the gut microbiota to promote switching IgM to IgA, collectively enhancing IgA levelsin critical factor for intestinal barrier integrity and pathogen defense (62). Liu et al. recently reported that *A. muciniphila* alleviates colitis by specifically expanding colonic lamina propria CD103^+^ DCs. These DCs specifically express retinaldehyde dehydrogenase 2 (RALDH2), a key enzyme for retinoic acid (RA) synthesis. The resulting RA facilitates the conversion of innate lymphoid cells from ILC1 to ILC3 and enhances production of IL-22, a cytokine crucial for mucosal repair (63).

### 2.2 The *A. muciniphila* and IECs

IECs not only selectively facilitate the uptake of nutrients but also serve as a physical and biochemical barrier that segregates the luminal microbial communities from the mucosal immune system, protecting the host from unnecessary immune responses to luminal contents or microbiota (64). In this section, we outline the bidirectional influence between *A. muciniphila* and IECs within IBD.

TLRs are present not only on the surface of immune cells but also on the surface of IECs. Shi et al. revealed that the live and pasteurized *A. muciniphila*, upon recognizing TLR2 in Caco-2 IECs, induced the activation of AMP-activated protein kinase (AMPK), which facilitated the assembly of tight junction proteins and contributed to the maintenance of the intestinal barrier integrity. Moreover, both live and pasteurized *A. muciniphila* inhibited NF-κB activation through the TLR2-mediated pathway and resulted in the alleviation of inflammatory disorders (65).

Other proteins present on the IECs have also shown associations with the abundance of *A. muciniphila* in IBD. The natural killer group 2 member D (NKG2D) receptor is a type of stimulatory immune receptor expressed on natural killer (NK) cells, γδ T cells, CD56^+^ T cells, CD8^+^ T cells, activated CD4^+^ T cells, etc. (66–69). The increased expression of NKG2D and NKG2D ligands is related to IBD pathogenesis through modulating T and innate immunity cell activity (66, 70–72). Several monoclonal antibodies targeting NKG2D have been tested and shown to have a positive effect in improving IBD (73–75). However, limited research has revealed the relationship between gut microbiota and NKG2D/NKG2D ligands in IBD. Hansen et al. discovered that the gut microbiota has an effect on the expression of NKG2D ligands on the small IECs. In particular, the abundance of *A. muciniphila*, whether increased by treating mice with vancomycin or decreased by treating mice with dietary xylooligosaccharides, was negatively correlated with the expression of NKG2D ligands (76). Indoleamine 2,3-dioxygenase 1 (IDO1) is the initial enzyme responsible for metabolizing tryptophan (Trp) in the Kynurenine (Kyn) pathway. The research of Alvarado et al. found that the IDO1-TG mice (transgenic mice characterized by the overexpression of fluorescence-tagged IDO1 in the IECs) exhibited a 3.0-fold higher abundance of *A. muciniphila* in comparison to wild-type mice. They demonstrated that overexpression of IDO1 in IECs promoted the differentiation of secretory cells and increased mucus production, leading to enhanced levels of mucin-associated microbiota (*A. muciniphila* and *Mucispirillum schaedleri*), ultimately resulting in the amelioration of colitis severity (77). It is more likely that *A. muciniphila* could be regulated by IDO1 on IECs, thereby effectively alleviating intestinal inflammation in IBD.

Autophagy is an important catabolic recycling pathway that degrades cytoplasmic materials by the lysosome to maintain cellular homeostasis. The interplay between autophagy and the gut microbiota plays a vital role in IBD (78). Currently, emerging evidence suggests that variations in autophagy-related genes of IECs could influence the abundance of *A. muciniphila*, thereby playing a significant role in IBD. Naama et al. constructed the constitutive activation of autophagy mice through mutating the phenylalanine residue at position 121 of the autophagy-initiating protein Beclin 1 to alanine (Becn1^F121A^ mice). The results indicated that continuous activation of autophagy protected the intestine from colitis, characterized by alleviating endoplasmic reticulum (ER) stress in goblet cells, thereby facilitating mucus secretion and altering the gut microbiome, specifically increasing *A. muciniphila* (79). Yang et al. discovered that the intestinal epithelium-specific autophagy-related 5 knockout (Atg5^-/-^) mice with a disruption of autophagic flux in the IECs dramatically decreased the abundance of *A. muciniphila* and *Lachnospiraceae* family compared with that of wild-type mice. Differential gene expression analysis revealed that two key IBD-related transcription factors, RORC and TBX21, were upregulated in Atg5^-/-^ mice (80).

Conversely, *A. muciniphila* can also modulate the autophagy of IECs. Yu et al. discovered that the supplementation of *A. muciniphila* regulated the intestinal microbiota and metabolites, activated the NLRP6 inflammasome, promoted autophagy of IECs, and maintained the normal secretory function of Paneth cells and goblet cells (57, 58). Wang et al. discovered that the outer membrane vesicles (OMVs) of *A. muciniphila* not only could restore the disturbed gut microbiota homeostasis by selectively promoting the proliferation of beneficial bacteria, but also could enter the Peyer’s patches and the IECs to elicit the immune regulation and stimulate goblet cells to produce mucus for repairing the mucus barrier (62).

### 2.3 The Interaction of *A. muciniphila* and ISCs

In the study of Duan et al., they assessed the regulatory relationship between fucose, gut bacteria, and ISCs. The research revealed that the administration of fucose promoted the proliferation of ISCs. However, ISCs were not directly impacted by fucose. They indicated that fucose administration-promoted ISCs-mediated intestinal epithelial development was gut microbiota-dependent, as confirmed by the administration of an antibiotic cocktail to the mice to eliminate their gut microbiota. Ultimately, they elucidated that fucose supplementation significantly increased the *A. muciniphila* and *A. muciniphila*-related propanoate metabolism. This process enhanced the stemness and function of ISCs via the Wnt signaling pathway (81). Kang et al. further confirmed that the secreted protein Amuc_1409 of *A. muciniphila* could promote the proliferation, regeneration, and epithelial development of ISCs by interacting with E-cadherin. This interaction leads to the dissociation of the E-cadherin/β-catenin complex, ultimately activating the Wnt/β-catenin signaling pathway (82).

## 3 The *A. muciniphila* and metabolism

**Figure 3:**
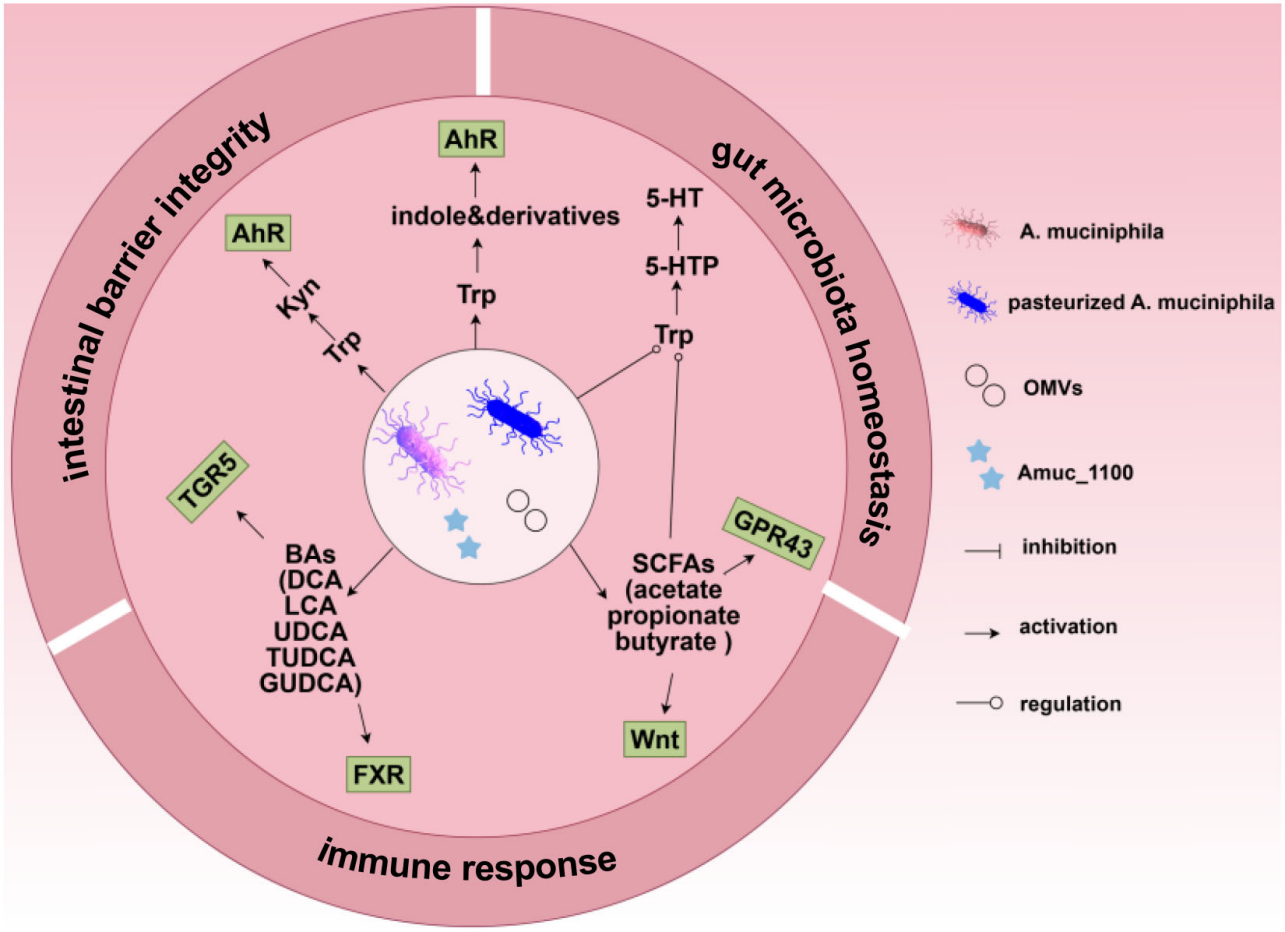
Live and pasteurized *A. muciniphila*, its component Amuc_1100, and its OMVs orchestrate gut homeostasis by modulating key metabolic pathways. They regulate the Trp/AhR pathway to promote anti-inflammatory cytokines, antimicrobial peptides, and microbiota for tissue repair; engage the SCFAs/GPR43 pathway to expand Treg cells and inhibit pro-inflammatory mediators; activate the SCFAs/Wnt pathway to enhance ISCs’ function and epithelial development; stimulate the BAs/FXR pathway to suppress inflammation and preserve mucin-secreting goblet cells; and activate the BAs/TGR5 pathway to promote epithelial repair via ISCs proliferation. Collectively, these integrated actions modulate intestinal immunity response, reinforce the intestinal barrier integrity, and reshape the gut microbiota homeostasis to alleviate IBD. Acronyms in the figure: Amuc_1100: an outer membrane protein of *A. muciniphila*; OMVs: *A. muciniphila* derived outer membrane vesicles; Trp: tryptophan; Kyn: kynurenine; AhR: aryl hydrocarbon receptor; 5-HT: 5-hydroxytryptamine; 5-HTTP: 5-hydroxytryptophan; SCFAs: short-chain fatty acids; GPR43: G-protein coupled receptors 43; Wnt: Wnt protein; BAs: Bile acids; DCA: deoxycholic acid; LCA: lithocholic acid; UDCA: ursodeoxycholic acid; TUDCA: tauroursodeoxycholic acid; GUDCA: glycoursodeoxycholic acid; TGR5: G protein bile acid receptor; FXR: farnesoid X receptor.

The imbalanced gut microbiota is associated with multiple human diseases due to its profound influence on essential physiological functions, which are regulated through direct cell-to-cell interactions or indirect metabolite-mediated pathways. In the interactions between the host and gut microbiota, a multitude of metabolic processes are involved, with a primary focus on tryptophan (Trp) metabolism, short-chain fatty acids (SCFAs) metabolism, and bile acids (BAs) metabolism. (**Figure 3**).

### 3.1 The *A. muciniphila* and Trp metabolism

Trp is an indispensable aromatic amino acid. Humans primarily acquire it from common natural foods. The metabolic activity of Trp takes place within the host cells and intestinal microbiota, mainly relying on the following three Trp metabolic pathways (83). Approximately 95% of Trp in the human body could be converted into kynurenic acid, quinolinic acid, nicotinic acid, and other compounds through the catalytic action of specific enzymes, which is known as the kynurenine (Kyn) pathway (84). Trp also could be transformed into indoles and indole derivatives. This process is known as the indole pathway. Additionally, through the catalysis of Trp hydroxylase and decarboxylase, Trp could be converted into 5-hydroxytryptamine (5-HT, also known as serotonin), and this is the 5-HT pathway (83). Many of the Trp metabolites, e.g., Kyn and indole, have been reported to be able to bind and activate the aryl hydrocarbon receptor (AhR). This entire process is termed the Trp-AhR pathway (85). Supplementation with Trp or several microbial Trp metabolites has been demonstrated to protect against colitis in mice through the activation of AhR, followed by the regulation of intestinal barrier, immune response, gut microbiota homeostasis, and other factors (86, 87). Recent studies have uncovered a mutual regulatory interplay between *A. muciniphila* and Trp metabolism in IBD.

The synthesis of 5-HT from Trp primarily involves two stages. Initially, Trp is transformed into 5-hydroxytryptophan (5-HTP) through the catalysis of Trp hydroxylase with indole ring hydroxylation. And then, 5-HT is produced undergoing the decarboxylation of 5-HTP, which takes place by Trp decarboxylase (83, 88). 5-HT was predominantly generated by enterochromaffin cells which are a subset of enteroendocrine cells in the gastrointestinal tract. The intestine holds approximately 95% of the total 5-HT levels in the body (89, 90). The research conducted by Yaghoubfar et al. demonstrated that *A. muciniphila* and its extracellular vesicles notably boosted 5-HT levels in colon tissue, increased the expression of serotonin transporter (SERT), and decreased the level of 5-HT in serum. Additionally, the OMVs of *A. muciniphila* had more biological effects on increasing the serotonin level in colon compared to the bacterium itself. Interestingly, the oral administration of *A. muciniphila* or its OMVs to male C57BL/6J mice without colitis for 4 weeks caused no adverse effect on inflammation, as there were no indications of inflammatory cell infiltration in the mucosal, epithelial, lamina propria, or submucosal layers (91). Several studies have shown that SCFAs, the metabolites of gut microbiota, play a crucial role in the production of 5-HT in the colon by promoting the expression of Trp hydroxylase 1 on enterochromaffin cells which is the rate-limiting enzyme for mucosal 5-HT synthesis (90, 92). *A. muciniphila* can metabolize mucin from the intestine with the production of SCFAs. Hence, the regulatory mechanism of *A. muciniphila*-SCFAs in Trp metabolism upon the 5-HT pathway remains to be elucidated in the future, especially concerning the amelioration of IBD.

In individuals with UC, there was a noted decrease in serum Trp levels and an activation of the Kyn pathway (93). Gu et al. comprehensively examined the impact of *A. muciniphila* on three Trp metabolic pathways in both UC patients and colitis mice through fecal metagenomics, untargeted serum metabolomics, and colonic transcriptomics. They confirmed that in UC patients or colitis mice, the Trp metabolism was activated with significantly decreased levels of Trp and related metabolites, such as Kyn, in the serum. Through treatment with live *A. muciniphila*, pasteurized *A. muciniphila*, or Amuc_1100, Trp metabolism was normalized by gut microbiota, exhibiting the restoration of Trp levels and a reduction in Kyn degradation. In addition, the expression of AhR targeted genes (*CYP1A1*, *IL-10*, and *IL-22*) was enhanced in colon of colitis mice through treatment with pasteurized *A. muciniphila* or Amuc_1100. But live *A. muciniphila*, pasteurized *A. muciniphila*, or Amuc_1100 failed to restore the decreased metabolites in the serotonin pathway during the colitis process. This indicated that from the perspective of regulating Trp metabolism, *A. muciniphila*, pasteurized *A. muciniphila*, or Amuc_1100 predominantly alleviated colitis in mice by inhibiting the Kyn pathway, activating the indole pathway, and enhancing AhR signaling. AhR, widely expressed in intestinal immune and epithelial cells, promotes the secretion of IL-10 and IL-22—L-22tionl that respectively prevent pro-inflammatory responses and protect against tissue damage by regulating antimicrobial peptides and microbiota (36). Although these studies suggested that *A. muciniphila* can simultaneously influence multiple pathways of tryptophan (Trp) metabolism in alleviating IBD, the in-depth mechanism of how *A. muciniphila* influences Trp metabolism in different pathways remains unclear. In addition, the influence of *A. muciniphila* on the state of the serotonin pathway with/without intestinal colitis remains inconclusive.

### 3.2 The *A. muciniphila* and SCFAs metabolism

The SCFAs, mainly acetate, propionate, and butyrate, are one of the metabolites through fermenting unabsorbed dietary fibers, non-starch polysaccharides, undigested proteins, as well as some peptides by gut microbiota. SCFAs play a pivotal role in host homeostasis, e.g., strengthening the gut barrier, regulating energy metabolism, and exerting immunomodulatory functions (94, 95). IBD patients exhibit a reduction in the levels of dominant SCFAs-producing microorganisms both in the intestinal mucosa and feces. Additionally, the concentrations of SCFAs in IBD patients seem to be lower compared with healthy individuals (96). *A. muciniphila* is a bacterium that is recognized for its ability to produce SCFAs, primarily acetate and propionate, by degrading mucin with its abundant enzymes. Recently, numerous studies have investigated the connection between *A. muciniphila*, SCFAs, and IBD.

*A. muciniphila* supplementation resulted in changes to the SCFAs profile. Bian et al. demonstrated that *A. muciniphila* exhibited protective effects on DSS-induced colitis mice. The pre-treatment of *A. muciniphila* to colitis mice not only stabilized the colon mucosal barrier, regulated inflammatory cytokines (e.g., TNF-α, IL-6) and chemokines MIP-1, but also alleviated dysbiosis of the gut microbiome. Remarkably, the *A. muciniphila* pre-treatment in the DSS-induced colitis mice resulted in a marked increase in SCFAs, including acetate, propionate, iso butyrate, and butyrate (30).

Although studies have shown that elevating the levels of *A. muciniphila* is beneficial for alleviating intestinal inflammation in mice, Zhai et al. discovered that there are strain-specific differences in improving intestinal colitis. They noticed that both *A. muciniphila* strains (strain ATCC BAA-835 and strain 139) contributed to the recovery of chronic colitis and promoted normalization of the gut microbiota in mice, but the ATCC BAA-835 strain exhibited a more significant effect. They further identified that the Treg cell differentiation and the production of SCFAs are the primary distinguishing factors in the effectiveness of the two strains. Only strain ATCC BAA-835 increased the overall concentration of SCFAs in the cecum with a significant upregulation of the G-protein coupled receptors 43 (GPR43) in colon and the number of Foxp3^+^ Treg cells in the mesenteric lymph nodes of mice. The possible mechanism underlying the beneficial effects of *A. muciniphila* in colitis is the upregulation of SCFAs. These SCFAs activate GPR43 and further regulate immune responses by increasing the number of colonic Foxp3^+^ Treg cells and inhibiting the expression of pro-inflammatory mediators (97).

Besides the *A. muciniphila* supplement directly, some other measures can alter the composition and abundance of *A. muciniphila* as well as the SCFAs level. Huang et al. found that the colon inflammation in DSS-induced mice could be significantly mitigated via lithium carbonate administration which is predominantly utilized in clinical settings as the antimanic and antidepressant drug. Notably, the administration of lithium carbonate not only changed the structure and composition of gut microbiota, especially resulting in an increased abundance of *A. muciniphila*, but also significantly transformed SCFAs profiles with a pronounced upregulation of acetate and propionate. They corroborated that *A. muciniphila* and its metabolic SCFAs, especially acetate and propionate, played a pivotal role in the improvement of colitis in lithium carbonate treatment. The SCFAs-sensing GPR43 was activated, which upregulated the population of Treg cells (CD25^+^Foxp3^+^CD4^+^) in colonic lamina propria and initiated anti-inflammatory responses (98). Duan et al. revealed that fucose treatment led to changes in the composition and functions of gut bacteria, with significant increases observed in *A. muciniphila* and its related propanoate metabolism. The acetate and propionate are the main SCFAs involved in propanoate metabolism of *A. muciniphila*. The increase in *A. muciniphila* and propanoate metabolism-related SCFAs triggered the activation of the Wnt signaling pathway, ultimately enhancing ISCs’ function and preserving the intestinal epithelium development (81). Luan et al. found that the ethanol extract of R. sterilis S. D. Shi fruits has the potential to ameliorate inflammation in DSS-induced mice. This effect was primarily characterized by the promotion of mucin expression, the inhibition of inflammatory mediator expression, the regulation of gut microbiota composition (notably the suppression of *Escherichia-Shigella* and the increase of *A. muciniphila*), and the enhancement of SCFAs production, particularly acetate (99).

Lee et al. carried out a study in which they pre-treated DSS-induced colitis mice with SCFAs, either sodium butyrate or a mixture of acetate, butyrate, and propionate, and then investigated the impact of SCFAs administration on intestinal inflammation and microbiota composition. During the experimental period, the mice were orally given SCFAs in their drinking water for 3 weeks, and in the third week, the mice were induced to have colitis through drinking water containing 2.0% DSS. The results determined that the oral administration of SCFAs did not yield a significant alleviation of colon inflammation. Supplementing with SCFAs affected T cell differentiation, resulting in enhanced expression of CD4^+^Foxp3^+^ regulatory T cells, which play a role in preventing excessive immune responses. Paradoxically, this supplementation also promoted the population of IL-17-producing T cells, exacerbating colon inflammation. In addition, the structure of gut microbial community was also altered in the SCFAs-treated group, which showed an elevated relative abundance of protective *A. muciniphila* and aggressive *Escherichia fergusonii* resulting in a neutral effect on colon inflammation in the DSS-induced colitis model. Taken together, these findings suggest that supplementing SCFAs alone has a neutral effect on colon inflammation (100).

### 3.3 The *A. muciniphila* and BAs metabolism

Primary BAs, e.g., cholic acid (CA) and chenodeoxycholic acid (CDCA), are small molecules that are produced in the liver from cholesterol. In hepatocytes, primary BAs are conjugated with either glycine or taurine, and conjugated BAs are subsequently released into the intestine. Approximately 95% of intestinal BAs are reabsorbed from the intestinal lumen and transported back to the liver through the portal vein. While the residual 5% are transported into colon, where they are either converted into unconjugated or secondary BAs (e.g., ursodeoxycholic acid (UDCA), lithocholic acid (LCA), deoxycholic acid (DCA)), or excreted in feces. The outcome depends on a variety of enzymes present in the colonic microbiota, e.g., bile salt hydrolase (BSH) and bile acid-inducible enzymes (BAI) (101–104). Abnormal synthesis or metabolism of BAs is related to the occurrence of diseases. Studies have revealed that patients with IBD and colitis animal models both exhibited abnormal BAs metabolism. And regulating BAs metabolism, such as targeting BAs receptors, has been employed as a therapeutic approach for IBD or colorectal cancer (105–110). The mutual interactions between gut microbiota and BAs metabolism in IBD have drawn significant attention from researchers. A portion of probiotics, e.g., *Bifidobacterium*, *Lactobacillus*, have exhibited the positive effects on regulating the gut microbiota-BAs axis (103). Recently, researchers have provided evidence of the regulatory relationship between *A. muciniphila* and BAs metabolism in IBD.

Supplementing with secondary BAs has been shown to increase the levels of *A. muciniphila* in feces and protect against colitis in mice. According to a study by Bossche et al., the UDCA and its taurine conjugates (tauroursodeoxycholic acid, TUDCA) or glycine conjugates (glycoursodeoxycholic acid, GUDCA) equally lowered the severity of DSS-induced colitis mice. They identified that colitis mice administered with UDCA, TUDCA, or GUDCA normalized the *Firmicutes/Bacteroidetes* ratio and increased the abundance of *Clostridium cluster XIVa* and *A. muciniphila*, which are recognized to be significantly reduced in IBD patients (111).

Dong et al. demonstrated that the polyphenol dihydromyricetin (DHM) not only ameliorates colitis by restoring microbial balance, notably enriching *Lactobacillus* and *A. muciniphila*, but also increases the levels of CDCA and LCA in a gut microbiota-dependent manner. These BAs subsequently activate their respective receptors, with LCA serving as the natural ligand for the G protein bile acid receptor (TGR5) and CDCA acting as the natural agonist for the nuclear receptor farnesoid X receptor (FXR). The activation of TGR5 and FXR plays a vital role in maintaining intestinal homeostasis through two key mechanisms. FXR activation protects against colitis by inhibiting pro-inflammatory cytokine production and preserving goblet cells, whereas TGR5 signaling promotes epithelial repair by enhancing the proliferation of ISCs. Together, they coordinately uphold intestinal integrity and regulate immune responses (112). Zhai et al. indicated that supplementation with *Eucommia ulmoides* leaf extracts (ELE) alleviated colitis, with a significant increase in the abundance of Akkermansiaceae and Ruminococcaceae. They discovered that ELE resulted in a marked increase in the concentrations of DCA and TUDCA in the serum, which are positively associated with the presence of *Akkermansiaceae* and *unidentified_Ruminococccaceae*. In addition, the expression of TGR5, which significantly maintains the barrier function of IECs, was also upregulated in the colons. and the improvement in colitis was mediated through the gut microbiota-BAs-TGR5 axis (113). These suggest that further efforts are required to clarify the exact regulatory mechanisms of *A. muciniphila*-BAs, potentially offering a viable strategy to address IBD.

Polysaccharides and other substances have been found to improve intestinal inflammation, along with the increase or decrease in the abundance of *A. muciniphila*. Nevertheless, the specificity between these substances and *A. muciniphila*, as well as the full molecular mechanisms, remains to be fully researched and clarified (114–120).

## 4 The security of *A. muciniphila*

Although there has been extensive research on the utilization of *A. muciniphila* in animal models, the assessment of its safety in the clinic is limited (121, 122). In 2019, Depommier et al. conducted the initial utilization of *A. muciniphila* in humans through a randomized, double-blind, placebo-controlled trial with overweight/obese and insulin-resistant volunteers. Their findings indicated that the daily oral supplementation of 10^10^ A*. muciniphila* bacteria for three months, whether in live or pasteurized form, was both safe, well-received and improved several metabolic parameters (123). In 2021, the EFSA Panel on Nutrition, Novel Foods, and Food Allergens issued a scientific opinion on the safety of pasteurized *A. muciniphila* (strain ATCC BAA-835T) as a novel food. The Panel determined that the pasteurized *A. muciniphila* at a daily intake of 3.4ake^10^ cells is safe for the target population as long as the quantity of viable *A. muciniphila* remains under 10 cells/g in the novel food, which opened doors for its application into food supplements and medical nutrition (124).

An analysis of the WHO International Clinical Trials Registry Platform and relevant literature indicates that research on *A. muciniphila* has predominantly focused on the effects of its live and pasteurized forms on metabolic disorders, including overweight, obesity, and insulin resistance (125–127). A smaller number of clinical studies have explored its impact on muscle strength and respiratory symptoms (128, 129). However, the clinical trials assessing the gastrointestinal inflammation are presently restricted to the impact of *A. muciniphila* on irritable bowel syndrome (130). There are few experiments to demonstrate its safety in the treatment of IBD. Hence, further clinical research is essential to confirm the potential and safety of *A. muciniphila* in mitigating intestinal inflammatory diseases.

## 5 Conclusions

The pathways of *A. muciniphila* in mitigating IBD are mainly represented by the regulation of the immune response, gut microbiota homeostasis, and intestinal barrier integrity. The majority of these pathways involve the participation of intestinal cells and microbial metabolism. In the future, more research efforts should be devoted to explore the mechanisms of various components or different forms of *A. muciniphila* in regulating intestinal cells and metabolism by integrating multi-omics data. In addition, it is equally crucial to investigate how intestinal cells and metabolic factors influence the colonization and abundance of *A. muciniphila*. This will contribute to a more in-depth and detailed theoretical foundation for the therapeutic application of *A. muciniphila* in the treatment of IBD.

The role of *A. muciniphila* in intestinal inflammation is occasionally controversial. *A. muciniphila* generally exerts protective effects in healthy individuals or those with metabolic disorders (e.g., obesity and type 2 diabetes), but it may become pathogenic under specific conditions, such as a disrupted barrier, coexistence with pathogenic bacteria, and susceptible hosts. Furthermore, interspecies differences (e.g., between humans and mice) and strain-specific variations of *A. muciniphila* itself collectively determine whether it alleviates or exacerbates IBD. It seems that the effectiveness of *A. muciniphila* in alleviating intestinal inflammation varies among individuals. Currently, both live and pasteurized *A. muciniphila*, along with their components and secreted products, have demonstrated potential in alleviating IBD, yet these findings are still at the animal level. The *A. muciniphila* has potential as a next-generation probiotic for disease therapy, but significant safety issues must be resolved before developing clinically available products for IBD treatment.
